# Amniotic fluid metabolomics and biochemistry analysis provides novel insights into the diet-regulated foetal growth in a pig model

**DOI:** 10.1038/srep44782

**Published:** 2017-03-16

**Authors:** Jin Wan, Fei Jiang, Jiao Zhang, Qingsong Xu, Daiwen Chen, Bing Yu, Xiangbing Mao, Jie Yu, Yuheng Luo, Jun He

**Affiliations:** 1 Institute of Animal Nutrition, Sichuan Agricultural University, Chengdu 611130, Sichuan, People’s Republic of China; 2 College of Fisheries and Life Science, Dalian Ocean University, Dalian 116023, Liaoning, People’s Republic of China

## Abstract

Foetal loss and intrauterine growth restriction are major problems in mammals, but there are few effective ways in preventing it. Intriguingly, chitosan oligosaccharide (COS), a biomaterial derived from chitosan, can promote foetal survival and growth. Therefore, we have investigated how COS affects foetal survival and growth in a pig model. Fifty-two sows were divided into two treatment groups (n = 26) and fed either solely a control diet or a control diet that includes 100 mg/kg COS. Amniotic fluid and foetus samples from six sows that were of average body weight in each group were collected on gestation day 35. We applied a ^1^H NMR-based metabolomics approach combined with biochemistry analysis to track the changes that occurred in the amniotic fluid of pregnant sows after COS intervention. Maternal COS inclusion had enhanced (*P* < 0.05) the foetal survival rate and size at 35 days. COS supplementation had both increased (*P* < 0.05) SOD, CAT and T-AOC activities and elevated (*P* < 0.05) IL-10, IgG and IgM concentrations in the amniotic fluid. Moreover, COS had affected (*P* < 0.05) the amniotic fluid’s lysine, citrate, glucose and hypoxanthine levels. Overall, COS inclusion induced amniotic fluid antioxidant status and metabolic profiles modifications characterising improvements in foetal survival and growth in a pig model.

Chitosan oligosaccharide (COS), which is a depolymerised product of chitosan, holds great potential applications in the food, pharmaceutical, agricultural and environmental industries^1^,^2^,^3^. In particular, COS has potential applications as a dietary supplement or nutraceutical for animals^4^,^5^. Appreciation of the important role of COS in regulating mammalian foetal survival and growth rates has grown steadily in recent years^6^,^7^. For example, the foetal survival rate in sows after 35 days COS supplementation was elevated by approximately 13.0%^6^. As such, maternal COS supplementation provides an important breakthrough for developing strategies to reduce prenatal loss. Unfortunately, the underlying mechanisms responsible for COS-induced foetal survival and growth alterations are not clearly elucidated. Therefore, further exploration is necessary.

Pregnancy is associated with the onset of many adaptation processes that will likely change over the gestation course^8^. In particular, the metabolic composition of blood and amniotic fluid should reflect these biochemical dynamics. Amniotic fluid originates from maternal, foetal and placental tissues; therefore, its metabolic profile is the net result of metabolite synthesis/degradation, foetal maturation (particularly of the kidneys and lungs), and biochemical exchanges^9^,^10^. On this account, amniotic fluid recapitulates the physiological processes of foetal development, which makes it an extremely valuable material for foetal health diagnostics^11^,^12^. Nevertheless, no studies have focused on COS-induced amniotic fluid metabolism changes in sows. Hence, it is necessary to employ a robust technique to simultaneously quantify and identify a large number (in the range of hundreds to thousands) of molecules in amniotic fluid.

Currently, proton nuclear magnetic resonance (^1^H NMR)-based metabolomics is an established technique for studying complex biological samples (e.g., plasma, urine or amniotic fluid)^13^,^14^,^15^. Metabolomics exploits high-throughput analytical measurements to identify and quantify metabolites, allowing the description of the dynamic changes in phenotype and system homeostasis^16^. Amniotic fluid metabolic profiles can offer new insights to better understand the organ systems and biofunctions that contribute to foetal well-being during a normal pregnancy^17^,^18^. The great advantage of such an approach is that all metabolites (those present in high enough concentration in the biological sample) are measured simultaneously, and a pattern of several metabolites (metabolic profile) can be more informative than when measuring a single metabolite/analyte^19^. However, it should be emphasised that the use of metabolomics in prenatal medicine is currently still in its infancy^20^.

As indicated previously, pig is a critical model for humans to investigate diet-induced foetal growth alterations, because the metabolic features and cardiovascular systems of pigs and humans are similar and their organ sizes are proportional^21^,^22^. Metabolomics offers a novel strategy with which to determine changes in the metabolic endpoints of organisms’ physiological regulatory processes after specific nutritional interventions. Herein, we hypothesised that COS may change the metabolic profiles and biochemical parameters in amniotic fluid, thus enhancing foetal survival and growth. Taking the above into consideration, the present study examines an explorative metabolomic approach through ^1^H NMR spectroscopy combined with biochemistry analysis to test this hypothesis in a pig model.

## Results

### Foetal survival rate and size

As shown in Supplementary Table S1, sows in the COS group had a higher foetal survival rate (*P* < 0.05) on gestation day 35 compared to the CON group. Notably, the sow foetal size (crown-to-rump length) was elevated (*P* < 0.05) at 35 days of COS inclusion.

### Foetal dvelopment–related genes expression

**Figure 1:**
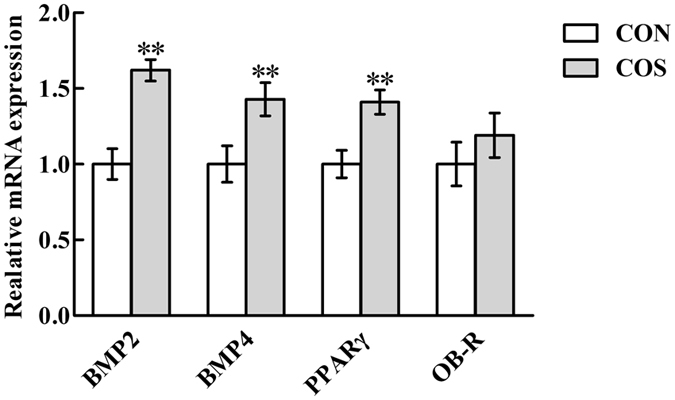
Effects of chitosan oligosaccharide supplementation on the relative mRNA expression of BMP2, BMP4, PPARγ and OB-R in the foetuses of sows. Values are means (six sows per treatment) with standard deviations represented by vertical bars. ^**^P < 0.01 (indicates that the relative mRNA expression in the COS group is significantly higher than that in the CON group). CON represents a corn–soybean basal diet; COS, chitosan oligosaccharide (represents the basal diet supplemented with 100 mg/kg chitosan oligosaccharide). BMP2, bone morphogenetic protein 2. BMP4, bone morphogenetic protein 4. PPARγ, peroxisome proliferator activated receptor γ. OB-R, obese receptor.

Foetal development**–**related gene (BMP2, BMP4, PPARγ and OB-R) mRNA expression levels in foetuses are presented in Fig. 1. In the foetuses, an increase (*P* < 0.05) in BMP2, BMP4 and PPARγ mRNA expression levels was observed in sows fed with COS-supplemented diets, compared to those in the CON group. However, there was no difference (*P* > 0.05) in the expression level of OB-R among the two treatment groups.

### Antioxidant indicators

The antioxidant parameters changes in the amniotic fluid observed after COS supplementation are listed in Table 1. Compared with the CON group, COS supplementation promoted increases (*P* < 0.05) in amniotic fluid SOD, CAT and T-AOC activities respectively by 14.62%, 44.60% and 23.48%. Meanwhile, noticeably increased (*P* < 0.05) ASA and AHR activities were found in the amniotic fluid after COS ingestion, and no changes (*P* > 0.05) of GSH and MDA content in the amniotic fluid were noted among the two groups.

### Immune parameters

Table 2 reveals data on the immune parameters of the sow amniotic fluid. Amniotic fluid concentrations of pro-inflammatory cytokines (IL-1, IL-6 and TNF-α) were not different (*P* > 0.05) between the two treatment groups. Meanwhile, the anti-inflammatory cytokines IL-10 concentration was significantly increased (*P* < 0.05) by COS supplementation. The amniotic fluid concentrations of IgG and IgM were respectively 64.06% and 29.74% higher (*P* < 0.05) in the COS-supplemented sows compared to the control sows. However, no significant difference (*P* > 0.05) was found in the amniotic fluid IgA concentration between the two treatments.

### ^1^H NMR spectra

**Figure 2:**
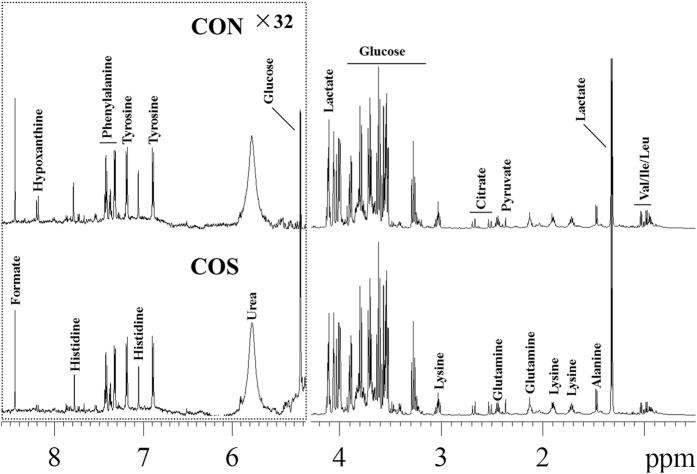
A representative example of the ^1^H NMR spectrum of amniotic fluid. Val represents valine, Leu is leucine and Ile is isoleucine. CON is a corn–soybean basal diet and COS is the basal diet supplemented with 100 mg/kg chitosan oligosaccharide.

Figure 2 shows the typical ^1^H NMR spectra of sow amniotic fluid from the CON group and the COS-treated group. In total, 16 metabolites were assigned in the amniotic fluid (Table 3). Moreover, the spectra of the amniotic fluid samples included resonances from lactate, citrate, pyruvate, glucose, formate, purine and amino acids.

### Multivariate data analysis of NMR data

**Figure 3:**
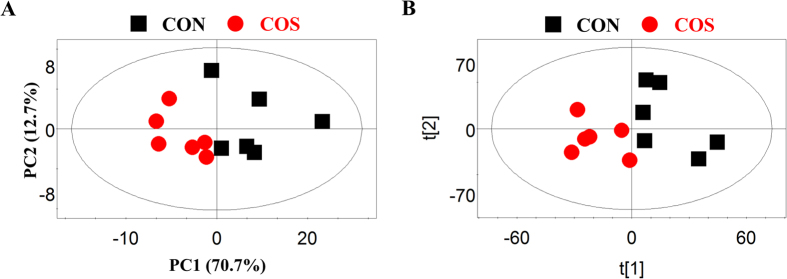
PCA (R^2^X = 83.4%, Q^2^ = 0.570; (**A**)) and PLS-DA (R^2^X = 58.9%, R^2^Y = 0.7867, Q^2^ = 0.417; (**B**)) score plots on the basis of the ^1^H NMR spectra of amniotic fluid samples from COS-treated (red circles) and CON groups (black squares). PCA, principal component analysis. PLS-DA, partial least squares discriminant analysis. CON is a corn–soybean basal diet and COS is the basal diet supplemented with 100 mg/kg chitosan oligosaccharide.

**Figure 4:**
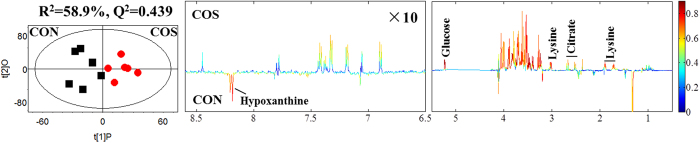
OPLS-DA score plots (left panel) and corresponding coefficient loading plots (right panel) obtained from the ^1^H NMR spectra of amniotic fluid samples from COS-treated and CON groups (R^2^X = 58.9%, Q^2^ = 0.439). The colour scale in the coefficient plot shows the significance of metabolite variation between the COS-treated and CON groups. OPLS-DA, orthogonal projection to latent structure with discriminant analysis. CON, a corn–soybean basal diet; COS, chitosan oligosaccharide (the basal diet supplemented with 100 mg/kg chitosan oligosaccharide).

PCA was initially performed on the amniotic fluid spectral data. Two principal components were calculated for the treatment groups, in which 70.7% and 12.7% of the variables were explained by PC1 and PC2, respectively. The PCA results (Fig. 3A) demonstrated that separation was not observed in the metabolic amniotic fluid profiles of the sows from either COS or CON groups. Thereafter, the amniotic fluid spectra of the COS and CON groups were subjected to PLS-DA (Fig. 3B). The score plots clearly highlighted two clusters that correspond to the two groups. Finally, the metabolic changes in the amniotic fluid of sows from the COS and CON groups were analysed by OPLS-DA. As per the corresponding coefficient analysis shown in Fig. 4 and Table 4, COS significantly increased (*P* < 0.05) the amniotic fluid lysine, citrate and glucose levels, but decreased (*P* < 0.05) the hypoxanthine level.

## Discussion

Amniotic fluid is the clear, watery liquid that surrounds growing foetuses within the amniotic cavity, and serves several important functions during foetal development^23^. However, oxidative stress can damage the normal amniotic fluid function through changes in certain substances^24^. Therefore, protecting the normal amniotic fluid functions from oxidative stress is indispensable in the maintenance of foetal development. Accordingly, we surveyed whether COS supplementation can mitigate oxidative damage in the amniotic fluid by evaluating the antioxidant-related parameters. COS ingestion significantly increased the representative enzymatic antioxidant activities (SOD and CAT) in the present study, suggesting that COS ingestion can partly enhance the function of antioxidant defence systems by improving enzymatic antioxidant activity^25^. A recent study reported that GSH and T-AOC are regarded as participants in non-enzymatic antioxidant defence systems^26^. Here, we found that dietary COS supplementation significantly elevated amniotic fluid T-AOC activity. As shown, these results further suggest that COS supplementation can enhance the sow amniotic fluid’s antioxidant capacity. ASA and AHR activities were respectively assessed to determine the total capacity of COS to scavenge superoxide anions and hydroxide radicals, both of which are strongly involved in cellular oxidative damage^27^. Remarkably, the present study showed that COS enhanced the superoxide-radical scavenging ability of sow amniotic fluid by improving ASA and AHR activities. Based on the above novel findings, we concluded that COS plays an important role in enhancing the amniotic fluid antioxidant defence properties.

It is now increasingly clear that the feto-placental unit survival and growth are influenced by a complex interactive network of cytokines, some of which are produced by local immune components and others by reproductive tissues^28^. Hence, we evaluated the effects of COS on the amniotic fluid inflammatory cytokines, including IL-1, IL- 6, IL-10 and TNF-α. Our results showed that dietary COS supplementation prominently increased the IL-10 level in the amniotic fluid. Chaouat *et al*. showed that anti- inflammatory cytokines such as IL-10 may have crucial roles in preventing feto-placental damage that could ensue from local inflammation^29^. As such, it is reasonable to speculate that COS supplementation initiated during early pregnancy can prevent foetal loss by increasing the IL-10 level in the amniotic fluid. Meanwhile, we also observed that amniotic fluid immunoglobulin levels (IgG and IgM) were enhanced by COS supplementation, which helps maintain optimal amniotic fluid immune status, and may be have a role in protecting the foetus against various virus infections^30^. However, the specific effects of enhanced amniotic fluid immunoglobulin levels exhibited by COS supplementation on foetal survival and growth remains largely unknown, making further investigation obligatory.

Maternal amniotic fluid metabolic profiles are valuable sources of information about foetal development, and can be potentially useful in the diagnosis of pregnancy disorders^31^. Therefore, a ^1^H NMR-based metabolomics approach has been applied to investigate COS-induced foetal survival and growth alterations in a pig model. In the current study, glucose, which is a major energy substrate and plays a role in animal growth and development^32^, was clearly increased in the amniotic fluid after COS supplementation. Therefore, COS can change the glucose metabolism in sows. An increased amniotic fluid glucose level also implies that carbohydrates and energy metabolisms have been altered. Hence, we presented that the intensive carbohydrates and energy metabolisms processes that may occur in sows during early pregnancy allow for rapid foetus growth via high carbohydrate and energy uptake through the placenta. These findings provide a strong scientific basis for supplementing COS for both pregnant sows and humans to improve foetal survival and growth.

Amino acids are a crucial part of a proper energy balance and in the maintenance of the tricarboxylic acid cycle span by providing carbon backbone exchange via various anaplerotic and cataplerotic pathways^33^. It is known that a shortage of amino acids can strongly influence foetal protein biosynthesis^34^. A previous study revealed that COS can promote placental amino acids transport from sows to foetuses^35^. At present, we have observed how COS supplementation can affect amino acid metabolism, and relative increases in the lysine level was found in the amniotic fluid of the COS group compared to the CON group. This is likely associated with foetal maturation and the increased demand for elementary building blocks, which are necessary for protein synthesis^36^ and might be utilised in many of the other processes required to maintain foetal homeostasis during rapid growth^34^. Citrate, which is synthesised in the mitochondrion, serves as a major precursor of cytoplasmic acetyl-CoA for the tricarboxylic acid cycle^37^, was significantly increased after COS intervention. Considering this observation, we hold the opinion that COS can enhance the tricarboxylic acid cycle, which is a nexus of the central carbon metabolism, elegantly balancing amino acids, carbohydrates and lipids metabolism^38^. Furthermore, it is worth noticing that citrate fluctuation in the amniotic fluid moderately corresponds to the glucose level, which was higher in the COS-treated sows compared to the control sows. Therefore, the sufficient citrate level was postulated to have an important role in foetal survival and growth maturation.

Maternal nutrition during gestation is closely associated with foetal growth and development in humans and animals, because it alters the foetal genome expression^39^,^40^. We next tested several foetal development related genes in foetuses to further our understanding of how maternal COS supplementation can affect foetal growth. Our present study provided the first evidence in a pig model that BMP2 and BMP4 mRNA levels in foetuses were increased by COS, which suggests that COS could partly mediate growth through improving growth factor levels. Furthermore, PPARγ, one of the marker genes responsible for adipogenesis^41^, was found to have increased when sows were fed a diet that included COS. In support of this view, we speculated that COS could facilitate foetal fatty acid *de novo* synthesis, which might partly account for COS’s growth-promotion property. In addition, proteomics studies must be performed to indicate changes in the foetal proteome after maternal COS inclusion.

In summary, COS supplementation could alter amniotic fluid antioxidant and immune status, in addition to the metabolic profiles, and thus create an optimal internal environment for foetal growth. Metabolic variations could be attributed to the functional variations in amino acid metabolism, glucose metabolism, the tricarboxylic acid cycle and oxidative protection, which have important practical implications in enhancing foetal survival and development. These novel findings might transfer to some extent into the clinical arena in the future.

## Methods

All experimental procedures in the present study were approved by the Animal Management Rules of the Ministry of Health of the People’s Republic of China and the Animal Care and Use Committee of Sichuan Agricultural University. We confirm that all methods were performed in accordance with the relevant guidelines and regulations.

### Animals

Fifty-two multiparous sows (Yorkshire; high-prolificacy gilts introduced to China from Canada), whose parities were in the range 3–4 were selected from a commercial pig farm (Leshan, China) and transported to Sichuan Agricultural University (Chengdu, China). The sows were individually housed in gestation crates (1.5 × 2.0 m) in a pregnancy room. The ambient temperature in the pregnancy room was maintained at 15–18 °C.

### Experimental design and diets

All sows were determined to be in the oestrous stage and were then inseminated twice with unfrozen semen via artificial insemination 3–5 days after weaning. The sows were randomly allotted to one of two treatments (26 sows/treatment) from day 1 of mating to ensure that each group had the same number of sows of similar parity. The treatment groups were as follows: (1) control diet without supplementation (CON); (2) control diet with COS added at a concentration of 100 mg/kg (COS).

The diets were formulated to meet or exceed the nutrient requirements recommended by the National Research Council (NRC) (2012)^42^, and their compositions are shown in Supplementary Table S2. COS was obtained from the Dalian Institute of Chemical Physics, Chinese Academy of Sciences (Dalian, China). The sows were fed twice daily either 2.2 kg of control or COS-supplemented diets during days 1 to 34 of gestation (at 08:00 and 18:00). In addition, all sows were given *ad libitum* access to water.

### Sample collection

At day 35 of gestation, 12 hours after their last meal, six sows of average body weight for each group were chosen. Thereafter, the selected sows were prepared for anaesthesia (15 min) and then hysterectomised to obtain conceptuses (foetuses and associated foetal membranes and fluids). Approximately 4 mL of amniotic fluid from each foetus was immediately collected for metabolomics and biochemical assays. Next, the foetal survival rate and size (crown-to-rump length) were recorded as previously described^43^ before collection and freezing at −80 °C for quantitative real-time polymerase chain reaction (qPCR). Finally, all amniotic fluid from the same sow was mixed and centrifuged at 2000× g for 10 min (at 4 °C) to remove meconium, and then stored at −80 °C before use.

### RNA extraction and reverse transcription

Total RNA was extracted from frozen foetal tissue (approximately 100 mg) with a TRIzol Reagent (Invitrogen, Carlsbad, CA, USA) according to a previous study^44^. The total RNA concentration was confirmed using a spectrophotometer (DU800, Beckman Coulter Inc., Brea, CA, USA) at 260 nm and 280 nm. RNA purity was determined by the absorption ratios (260/280 nm), which were 1.8–2.0 for all samples. RNA integrity was detected by 1% agarose gel electrophoresis. Two micrograms of total RNA were reversely transcribed into cDNA using a PrimeScript^TM^ RT Reagent kit (Takara Bio Inc., Dalian, China) according to the manufacturer’s instructions, and reverse transcription was performed at 37 °C for 15 min and 85 °C for 5 s in a Thermal Cycler PTC0200 (BioRad Laboratories, Hercules, CA, USA).

### qPCR

All primers were synthesised commercially by Invitrogen (Shanghai, China) and shown in Supplementary Table S3. qPCR was performed with the SYBR^®^ Green PCR I PCR reagents (Takara Bio Inc., Dalian, China) using a CFX96 Real-Time PCR Detection System (Bio-Rad Laboratories, Hercules, CA, USA). All foetal samples were detected in triplicate. The reaction mixture (10 μL) contained 5 μL of freshly SYBR^®^ *Premix Ex* Taq^TM^ II (Tli RNaseH Plus, 2×), 1 μL forward primers (4 μM) and 1 μL reverse primers (4 μM), 1 μL reverse transcription products and 2 μL nuclease-free water. The PCR conditions were pre-run at 95 °C for 10 s, and 40 cycles of denaturation steps at 95 °C for 5 s, followed by an annealing temperature of 55.7 °C for 30 s, and a 72 °C extension step for 10 s. After amplification, melting curve analysis was performed to confirm each product’s specificity. Melting curve conditions were 1 cycle of denaturation at 95 °C for 10 s and then 65 °C changed to 95 °C at a temperature change velocity of 0.5 °C/s. The standard curve of each gene was run in triplicate to obtain reliable amplification efficiency values. The correlation coefficients of all standard curves were >0.99, and the amplification efficiency values were 90–110%. *GAPDH* expression was used as a reference gene to normalise the mRNA expression of the target genes, and the relative quantification of gene expression among the treatment groups was analysed using the 2^−ΔΔCt^ method^45^.

### Determination of antioxidant parameters in the amniotic fluid

Amniotic fluid antioxidant status evaluation was performed using a microplate reader (SpectraMax M2, Molecular Devices, USA) that contained the malondialdehyde (MDA), superoxide dismutase (SOD), catalase (CAT), glutathione (GSH), anti-superoxide anion (ASA), anti-hydroxyl radical (AHR), and the total antioxidant capacity (T-AOC). All antioxidant-related kits were furnished by the Nanjing Jiancheng Bioengineering Institute (Nanjing, China).

#### MDA content analysis

MDA content was examined according to a procedure used by Livingstone *et al*.^46^. MDA content was assayed by reacting with thiobarbituric acid in an acidic medium for 30 minutes at 95 °C to generate a pink product that could be spectrophotometrically determined at 532 nm. MDA results were expressed in nmol per millilitre of amniotic fluid.

#### SOD activity analysis

SOD activity was measured spectrophotometrically at 550 nm using a method described in a previous study^47^. This technique involves decreasing the product (superoxide ions) of the xanthine/xanthine oxidase system and the formation of red formazan by reaction with 2-(4-iodophenyl)3-(4-nitrophenol)-5-phenyltetrazolium chloride. SOD activity was presented as U per millilitre of amniotic fluid, and 1 U of SOD would denote the 50% inhibition of superoxide ion production in the reaction.

#### CAT activity analysis

CAT activity was examined using the colorimetric method described by Özmen *et al*.^48^. The enzymatic reaction was terminated by the addition of ammonium molybdate, which generated a light-yellow composite that could be measured at 405 nm. CAT activity was expressed as U per millilitre of amniotic fluid, and 1 U of CAT is defined as the amount of enzyme needed to decrease 1 mmol/L of H_2_O_2_ at 37 °C for 1 s per millilitre of amniotic fluid.

#### GSH content analysis

GSH content was spectrophotometrically determined at 412 nm because the reaction between reduced GSH and 5,5′- dithiobis-p-nitrobenzoic acid can form yellow-colour 5-thio-2-nitrobenzoic acid, which is readily detectable^49^. GSH content in the extract was presented as mg per millilitre. In addition, commercial GSH was applied as standard.

#### ASA activity analysis

ASA was measured in accordance with the method of Jiang *et al*.^50^. O_2_^−^ was generated by the reaction of xanthine and xanthine oxidase. A colouration reaction is developed using the Griess reagent after the addition of the electron acceptor; the colouration degree is directly proportional to the amount of superoxide anion in the reaction.The amniotic fluid’s ASA capacity is expressed in U per litre of amniotic fluid, where one U is the quantity of superoxide anion free radicals scavenged within 40min per llitre of amniotic fluid, which is equal to each microgram of vitamin C–scavenging under the same conditions.

#### AHR activity analysis

AHR was measured in accordance with the method of Jiang *et al*.^50^. OH^−^ was generated on the basis of the Fenton reaction (Fe^2+^ + H_2_O_2_/Fe^3+^ + OH^−^ + ^•^OH). A coloration reaction is developed using the Griess reagent after the addition of the electron acceptor. The coloration degree is directly proportional to the quantity of hydroxyl radicals in the reaction. Amniotic fluid AHR capacity is expressed in U per millilitre of amniotic fluid, where one unit is defined as the amount that decreases 1 mmol/L H_2_O_2_ within 1 min per millilitre of amniotic fluid.

#### T-AOC activity analysis

T-AOC determination would enable the evaluation of the total activities of several parameters, including polyphenol complexes, protein thiol groups, glutathione and vitamins C and E, all of which can convert Fe^3+^ to Fe^2+^. Fe^2+^ can then be combined with phenanthroline to form stable and coloured chelates. T-AOC was estimated at 550 nm and expressed as U per millilitre of amniotic fluid. Here, 1 U represents the 0.01 increase in the absorbance value in 1 minute per millilitre of amniotic fluid.

### Amniotic fluid cytokines and immunoglobulins assay

All immune indices were analysed using commercially available porcine Enzyme-Linked Immunosorbent Assay kits purchased from R&D system (Minneapolis, MN, USA). Amniotic fluid cytokines (IL-1, IL-6, IL-10 and TNF-α) and immunoglobulin (IgA, IgG and IgM) concentrations were detected following manufacturer’s instructions. Cytokine and immunoglobulin concentrations were presented as pg/mL and μg/mL of amniotic fluid, respectively.

### Sample preparation and ^1^H NMR measurement

Aliquots of amniotic fluid were thawed at room temperature for 1 hour prior to the analysis. The amniotic fluid analysis was proceeded with 200 μL of amniotic fluid reconstituted into 400 μL of phosphate buffer (0.045 M NaH_2_PO_4_/K_2_HPO_4_, pH 7.4, 100% D_2_O). The mixture was then centrifuged at 16,000× g at 4 °C for 10 minutes, and 550 μL of the supernatant was transferred into 5 mm NMR tubes (Norell, Landisville, NJ, USA).

NMR spectra were recorded using an Agilent DD2 600 MHz NMR spectrometer (Agilent Technologies, Inc., CA, USA) operating at 599.93 MHz ^1^H at 298 K. A standard water-suppressed one-dimensional NMR spectrum was obtained for amniotic fluid using a standard NOESY pulse sequence (recycle delay −G1−90°−t_1_−90°−t_m_−G2−90°−acquisition) with a 2 s recycle delay and a 100 ms t_m_. The 90° pulse length was adjusted to about 10 μs for each sample and 64 transients were collected into 32,000 data points over a 20 ppm spectral width. Metabolite assignments were usually obtained by considering chemical shifts, coupling constants and relative intensities.

### ^1^H NMR spectroscopic processes and analysis

All free induction decays were multiplied by an exponential weighting function corresponding to a 1 Hz line-broadening before Fourier transformation. All ^1^H NMR spectra were corrected for phase and baseline distortions using Topspin 3.0 (Bruker Biospin). The chemical shifts in amniotic fluid spectra were referenced to the anomeric proton signal of α-glucose at δ 5.23.

Each amniotic fluid sample ^1^H NMR spectrum (δ 8.6–0.5) was automatically data reduced to 1700 integral segments of equal length (0.002 ppm) using MestReNova (Mestrelab Research). The water (5.17–4.28 ppm) and urea (6.50–5.50 ppm) regions were removed before data analysis to eliminate the effects of variation in the suppression of the water and urea signals. Integrated data were normalised to the total sum of the spectrum prior to principal component analysis (PCA), partial least squares discriminant analysis (PLS-DA) and orthogonal projection to latent structure with discriminant analysis (OPLS-DA) to give the same total integration value for each spectrum.

PCA was performed using SIMCA-P 11.5 (Umetrics, Sweden). PCA is an unsupervised pattern recognition method, and can be used for viewing “clusters” within multivariate data. Multivariate data can be displayed in a few principal components as a set of “scores” that highlight general trends and outliers. PLS-DA and OPLS-DA were performed using a unit variance-scaled approach. PLS-DA is a supervised pattern recognition method that explains the maximum separation between defined class samples in multivariate data. PLS-DA is performed by PLS regression against a specific cluster of samples as a dummy variable. Once a PLSDA model is calculated and validated, it can be used to predict class membership for unknown samples. OPLS-DA was performed with the NMR data to facilitate interpretation of loading. The model coefficients were back-calculated from the coefficients incorporating the weight of the variables and plotted with color-coded coefficients to enhance the model’s interpretability. Thus, the metabolites responsible for the differences between samples detected in the scores plot could be extracted from the corresponding loadings with the weight of the variable contributing to the discrimination. A correlation coefficient of r > 0.755 was the significance cut-off value based on the discrimination significance at *P* < 0.05, which was determined according to the significance test of the Pearson’s product–moment correlation coefficient.

### Statistical analysis

All amniotic fluid antioxidant and immune parameters, as well as foetal data (except the foetal survival rate), were statistically analysed using the Student’s *t*-test of SAS 9.0 (SAS Institute, Cary, NC, USA). The foetal survival rate data were analysed using a chi-square test within SAS. Each sow was considered as a statistical unit. Data are presented in the format mean ± standard deviation. *P* < 0.05 was considered significant when used to compare the differences between the two groups.

## Additional Information

**How to cite this article**: Wan, J. *et al*. Amniotic fluid metabolomics and biochemistry analysis provides novel insights into the diet-regulated foetal growth in a pig model. *Sci. Rep.* **7**, 44782; doi: 10.1038/srep44782 (2017).

**Publisher's note:** Springer Nature remains neutral with regard to jurisdictional claims in published maps and institutional affiliations.

## Supplementary Material

Supplementary Tables

## Figures and Tables

**Table 1:** Effects of chitosan oligosaccharide supplementation on the amniotic fluid antioxidant status of sows^a^.

Items	Treatments^b^		*P*-value
	CON	COS	
SOD^c^ (U/mL)	60.26 ± 4.80	69.07 ± 6.46^*^	0.023
CAT^d^ (U/mL)	3.61 ± 0.60	5.22 ± 0.53^**^	<0.001
ASA^e^ (U/L)	89.29 ± 1.86	104.68 ± 6.44^**^	0.005
AHR^f^ (U/mL)	398.23 ± 12.72	426.41 ± 14.15^*^	0.011
T-AOC^g^ (U/mL)	2.64 ± 0.16	3.26 ± 0.59^*^	0.048
GSH^h^ (mg/mL)	0.29 ± 0.06	0.31 ± 0.06	0.598
MDA^i^ (nm/mL)	4.90 ± 0.71	4.56 ± 0.56	0.386

^*^P < 0.05 versus the CON group. ^**^P < 0.01 versus the CON group.

^a^Values are means of six replications per treatment.

^b^CON: A corn–soybean basal diet; COS: Chitosan oligosaccharide (the basal diet supplemented with 100 mg/kg chitosan oligosaccharide).

^c^SOD: Superoxyde dismutase.

^d^CAT: Catalase.

^e^ASA: Anti-superoxide anion.

^f^AHR: Anti-hydroxyl radical.

^g^T-AOC: Total antioxidant capacity.

^h^GSH: Glutathione.

^i^MDA: Malondialdehyde.

**Table 2:** Effects of chitosan oligosaccharide supplementation on the amniotic fluid immune responses of sows^a^.

Items	Treatments^b^		*P*-value
	CON	COS	
IL-1^c^ (pg/mL)	163.33 ± 20.82	190.06 ± 49.46	0.250
IL-6^d^ (pg/mL)	34.19 ± 6.46	30.79 ± 5.36	0.345
IL-10^e^ (pg/mL)	109.67 ± 10.33	136.42 ± 15.36^**^	0.005
TNF-α^f^ (pg/mL)	142.36 ± 22.36	155.72 ± 15.78	0.259
IgG^g^ (μg/mL)	92.75 ± 21.92	152.17 ± 8.82^**^	<0.001
IgA^h^ (μg/mL)	64.23 ± 9.03	70.83 ± 10.40	0.268
IgM^i^ (μg/mL)	76.26 ± 19.60	98.94 ± 12.98^*^	0.040

^*^P < 0.05 versus the CON group. ^**^P < 0.01 versus the CON group.

^a^Values are means of six replications per treatment.

^b^CON: A corn-soybean basal diet; COS: Chitosan oligosaccharide (the basal diet supplemented with 100 mg/kg chitosan oligosaccharide).

^c^IL-1: Interleukin 1.

^d^IL-6, Interleukin 6.

^e^IL-10: Interleukin 10.

^f^TNF-α: Tumour necrosis factor α.

^g^IgG: Immunoglobulin G.

^g^IgA: Immunoglobulin A.

^i^IgM: Immunoglobulin M.

**Table 3:** ^1^H NMR data of metabolites in sow amniotic fluid^a^.

Keys	Metabolites	Moieties	δ^1^H (ppm) and multiplicity
1	Valine	γCH_3_, γ’CH_3_, βCH, αCH	0.98 (d), 1.04 (d), 2.27 (m), 3.61 (d)
2	Leucine	δCH_3_, δ’CH_3_, γCH, αCH_2_	0.95 (d), 0.96 (d), 1.69 (m), 3.73 (t)
3	Isoleucine	γCH_3_, δCH_3_, βCH, αCH	0.93 (t), 1.00 (d), 1.99 (m), 3.68 (d)
4	Lactate	βCH_3_, αCH	1.33 (d), 4.11 (q)
5	Alanine	βCH_3_, αCH	1.47 (d), 3.78 (q)
6	Lysine	γCH_2_, δCH_2_, βCH_2_, εCH_2_, αCH	1.49 (m), 1.70 (m), 1.89 (m), 3.01 (t), 3.76 (t)
7	Glutamine	αCH, βCH_2_, γCH_2_	2.13 (m), 2.44 (m), 3.76 (m)
8	Citrate	half CH_2_, half CH_2_	2.56 (d), 2.67 (d)
9	α-Glucose	1-CH, 2-CH, 3-CH, 4-CH, 5-CH, 6-CH	5.23 (d), 3.54 (dd), 3.71 (dd), 3.42 (dd), 3.84 (m), 3.78 (m)
	β-Glucose	1-CH, 2-CH, 3-CH, 4-CH, 5-CH, 6-CH, 6-CH’	4.66 (d), 3.26 (dd), 3.50 (t), 3.41 (dd), 3.47 (dd), 3.73 (dd), 3.90 (dd)
10	Tyrosine	3-CH & 5-CH, 2-CH & 6-CH	6.89 (d), 7.18 (d)
11	Urea	NH_2_	5.78 (s)
12	Histidine	4-CH, 2-CH	7.05 (s), 7.80 (s)
13	Phenylalanine	2-CH & 6-CH, 4-CH, 3-CH & 5-CH	7.31 (m), 7.37 (m), 7.42 (m)
14	Formate	H-COOH	8.45 (s)
15	Hypoxanthine	2-CH, 7-CH	8.22 (s), 8.20 (s)
16	Pyruvate	CH_3_	2.36 (s)

^a^s: Singlet; d: Doublet; t: Triplet; q: Quartet; dd: Doublet of doublets; m: Multiplet.

**Table 4:** OPLS-DA coefficients obtained from the NMR data of amniotic fluid metabolites from the COS and CON groups.

Metabolites	OPLS-DA coefficient (r)^a^
	COS versus CON^b^
Lysine	+0.773
Citrate	+0.798
Glucose	+0.868
Hypoxanthine	−0.806

^a^Metabolite keys are shown in Table 3; correlation coefficients were calculated from the OPLS-DA results with positive and negative signs respectively indicating positive and negative concentration correlations. OPLS-DA: Orthogonal projection to latent structure with discriminant analysis.

^b^CON: A corn-soybean basal diet; COS: Chitosan oligosaccharide (the basal diet supplemented with 100 mg/kg chitosan oligosaccharide).
