# Supplementary material for: Amniotic fluid metabolomics and biochemistry analysis provides novel insights into the diet-regulated foetal growth in a pig model
Source: Sci Rep. 2017 Mar 16;7:44782. doi: 10.1038/srep44782 (PMC5353717; doi:10.1038/srep44782)
Supplement: Supplementary Tables [file srep44782-s1.doc]

**Amniotic fluid metabolomics and biochemistry analysis provides novel insights into the diet-regulated foetal growth in a pig model**

Jin Wan,^1^ Fei Jiang,^1^ Jiao Zhang,^1^ Qingsong Xu,^2^ Daiwen Chen,^1^ Bing Yu, ^1^ Xiangbing Mao,^1^ Jie Yu,^1^ Yuheng Luo^1^ and Jun He^*1^

^1^ Institute of Animal Nutrition, Sichuan Agricultural University, Chengdu 611130, Sichuan, People’s Republic of China

^2^ College of Fisheries and Life Science, Dalian Ocean University, Dalian 116023, Liaoning, People’s Republic of China

J. Wan: [wanjin91@163.com](mailto:wanjin91@163.com)

F. Jiang: 309556494@qq.com

J. Zhang: zjpm25@163.com

Q.S. Xu: [xuqingsong@dl](mailto:xuqingsong@dl)ou.edu.cn

D.W. Chen: dwchen@sicau.edu.cn

B. Yu: ybingtian@yahoo.com.cn

X.B. Mao: acatmxb2003@163.com

J. Yu: jerryyujie@163.com

Y.H. Luo: luoluo212@126.com

J. He: [hejun8067@163.com](mailto:hejun8067@163.com)

*Running title: Chitosan oligosaccharide enhances sow metabolism*

^*^Corresponding author, Professor Jun He, Mobile, +86-13419354223, Fax: +86-28-86290920, e-mail: hejun8067@163.com.

**Table S1. Effects of chitosan oligosaccharide on the foetal survival rate and size of sows at gestation day 35**

| Items | Treatments^a^ | | *P*-value |
| --- | --- | --- | --- |
|  | CON | COS |  |
| Foetal survival rate^b^ (%) | 53.80 | 66.39^*^ | 0.031 |
| Foetal size^c^ (cm) | 3.83 ± 0.07 | 4.16 ± 0.06^**^ | <0.001 |

^*^ *P*<0.05 versus the CON group. ^**^ *P*<0.01 versus the CON group.

^a^CON: A corn-soybean basal diet; COS: Chitosan oligosaccharide (the basal diet supplemented with 100 mg/kg chitosan oligosaccharide).

^b^Foetal survival rate (%) = viable foetuses/ovulation rate×100

^c^Foetal size was considered as crown-to-rump length.

**Table S2. Composition and calculated nutrient content of the basal diet**

| Ingredient | Content (%) |
| --- | --- |
| Corn (7.8% crude protein) | 61.35 |
| Soybean meal ( 44.2% crude protein) | 13.20 |
| Wheat bran | 18.80 |
| Fish meal (62.5% crude protein) | 1.50 |
| Mono-calcium phosphate | 1.50 |
| Limestone | 0.95 |
| Salt | 0.50 |
| _L_-Lysine HCl (78%) | 0.20 |
| Vitamin-mineral premix^a^ | 2.00 |
| Calculated composition |  |
| Digestible energy (MJ/kg) | 12.55 |
| Crude protein | 14.51 |
| Lysine | 0.61 |
| Calcium | 0.95 |
| Total phosphorus | 0.81 |
| Available phosphorus | 0.53 |

^a^ The premix provided the following per kg of diets: 11023 IU vitamin (V) A, 1653.45 IU VD_3_, 44.09 IU VE, 4.4 mg menadione, 24 μg VB_12_, 9.9 mg riboflavin, 33 mg pantothenic acid, 55.1 mg niacin, 1551.0 mg choline, 0.22 mg biotin, 1.7 mg folic acid, 15.2 mg pyridoxine, 165.3 mg Zn, 39.7 mg Mn, 165.3 mg Fe, 16.5 mg Cu, 3.0 mg I and 3.0 mg Se.

**Table S3.** Primers used for quantitative real-time PCR

| Genes | Accession no. | Primers sequence (5′-3′) | Annealing temperature (°C) |
| --- | --- | --- | --- |
| BMP2^a^ | HQ110078.1 | F: TGTGGGCTGGAATGACTGG  R: GCACGCCTTTGGGATCTTAG | 60.0 |
| BMP4^b^ | NM001101031.1 | F: GAGGGCTCGGAAGAAGAATAA  R: GGTCAGCCAGTGGAAAGG | 60.0 |
| PPARγ^c^ | DQ437885.1 | F: CCAGCATTTCCACTCCACACTA  R: GACACAGGCTCCACTTTGATG | 59.0 |
| OB-R^d^ | GQ268934.1 | F: GCAGCGTGAGAAGTTATGTGG  R: TAAAGGATAAGCACTGAGCGACT | 60.0 |
| GAPDH^e^ | NM001206359.1 | F: ATGGTGAAGGTCGGAGTGAAC  R: CTCGCTCCTGGAAGATGGT | 59.0 |

^a^ BMP2: Bone morphogenetic protein 2.

^b^ BMP4: Bone morphogenetic protein 4.

^c^ PPARγ: Peroxisome proliferator activated receptor γ.

^d^ OB-R: Obese receptor.

^e^ GAPDH: Glyceraldehyde-3-phosphate dehydrogenase.
